# On Insufficient Alimentation, and the Value of Phosphate, &c.

**Published:** 1854-05

**Authors:** 


From the Virginia Medical and Surgical Journal.

*On Insufficient Alimentation, and the Value of Phosphate of Lime in Nutrition.*

In the *Bulletin de l' Acadamie Imperiale de Medicine*, for January, 1854, we find a report by M. Bouchardat, on the researches of a young and learned chemist, M. Mouries, in regard to the effects of phosphate of lime in the nutrition of animals, and the influence which the judicious employment of this salt is capable of exercising upon the mortality of children in large cities.

It has been a comparatively short period since physiologists began to appreciate properly the importance of inorganic principles in the phenomena of life. The farther we penetrate into this complex problem, the greater is the importance attributed to bodies, the presence of which in the human organism was regarded as quite accidental.

Very dissimilar organic compounds may be substituted for each other in our diet without any disorder in the general harmony, but the inorganic principles can only be replaced by substances very closely analagous to them. Albumen, fibrin, and casein, and other more complex aliments, though differing in origin and composition, may fulfil the same physiological end, but it is different with inorganic principles. Lecanu has shown that iron is indispensable for the proper constitution of blood-globules; chloride of sodium is of primary importance also as a constituent of the liquor sanguinis, and it is only as an exception that we find, in certain gramnivora, this salt partially replaced by the phosphate of soda or of potash. Liebig has shown that the chloride of potassium of the muscles cannot be replaced by chloride of sodium. Each inorganic constituent of the organism has, therefore, its definite and limited sphere of action, to which it is exclusively adapted.

Among the indispensable inorganic salts, the phosphate of lime holds an important rank. M. Mouries has devoted himself to the elucidation of its peculiar action. He deduces from his experiments the following conclusions:

1. Phosphate of lime plays a more important part in nutrition than has heretofore been believed. Independently of its necessity as a constituent of bone, this salt maintains that irritability without which there is no assimilation, and consequently no nutrition. Its insufficiency, therefore, produces death with all the symptoms of inanition, while its insufficiency in a less degree, produces a series of lymphatic diseases.

2. The food consumed in cities is deficient in this respect. Nurses' milk has, consequently, the same defect. The infant as well as the fœtus suffers from the deprivation of this element so indispensable to its development and life. Hence one of the causes of the increase in the number of still-born children, and of the mortality of infancy.

3. The addition of this salt, in combination with animal matter, to alimentary substances, obviates one cause of disease and death.

The following are the principal facts on which M. Mouries relies to establish these conclusions:

The blood of animals contains a constant proportion of earthy phosphates, which is independent of their ingesta. The pigeon ingests phosphate of lime slightly in excess, in the grain and calcarious gravels which it picks up; the horse swallows an excess, in its fodder; the dog procures a still greater excess from the bones on which he is fed; and y t the blood of the pigeon contains in

1000 grammes 1.20 of phosphate of lime; the horse 0.5; the dog 0.4. This result is not accidental; all birds whose blood has been analyzed have 1.5 to 1.2 of phosphate of lime, while the proportion in the blood of the carnivora and herbivora varies from 0.9 to 0.4. The proportion thus regulated by nature, is modified by age and sex. The bull, cow, and calf have the same food, yet their blood contains respectively 0.5 0.9, 0.8 of phosphate of lime.

The requisite proportion of alkaline phosphates varies, therefore, in different animals. A pigeon weighing one pound died at the end of ten months, during which period he was fed daily on one ounce of wheat, with common water for a drink, by which rather more than a grain of phosphate of lime was ingested daily: on the other hand, a woman weighing 100 pounds enjoyed perfect health upon a diet which furnished her daily with 90 grains of phosphate of lime. Thus health in the one case, and death, in the other, with relatively equal quantities of this salt.

We shall recur to this example to show how complex are the conditions of these experiments, and what reserve is necessary in drawing conclusions from them.

M. Mouries asserts, and the fact has already been noted by Chossat, that if the proportion of alkaline phosphates of the food is deficient, there ensues atony of the digestive organs, imperfect assimilation, and death. To prove that pigeons die from want of phosphate of lime, we may observe that their death is hastened if they are allowed only distilled water, while their lives may be preserved by adding earthy phosphates to their food.

M Bouchardt observed that the grain on which MM. Mouries and Chossat fed their pigeons contained only traces of common salt The birds therefore should be expected to suffer from the deprivation of this principle. M. Bouchardt accordingly made this experiment; he confined two pigeons, and fed them on dried grain. In two months the health of the female became impaired; she suffered from thirst and diarrhœa and laid no more eggs. She was set at liberty. She flew immediately to a window-sill impregnated with alkaline chlorides, and began to peck eagerly; there was a larger quantity of salts on the interior of the window-frame; the pigeon entered through the open window, and permitted herself to be re-captured, so imperious was her demand for these principles. Her health was re-established; in three days she laid another egg. It is wrong, therefore, to conclude with M. Mouries that a deficiency of phosphates is the only cause of the symptoms he observed; in this case, the absence of chlorides was the obvious cause.

M. Mouries has established, by interesting calculations, that grain furnishes a sufficient supply of phosphate of lime for the reparation of bone, but not for other essential functions of the economy. From the curious fact that there is a constant proportion between the temperature of animals, and the amount of phosphate of lime contained in their blood, he deduces the principle that this salt keeps up animal irritability, without which nutrition is impossible. The following table must interest physiologists:

| | Phosphate of Lime. Mouries | Poggiale. | Temperature. |
|---|---|---|---|
| Blood of the duck, - | 1.50 | | 42°5 cent. |
| ——— the hen, - | 1 35 | 1.25 | 41°5 " |
| ——— the pigeon, - | 1 20 | 1.23 | 40° " |
| ——— man, - | 0.80 | 0.6 | 37°5 " |
| ——— horse, - | 0.40 | 0.5 | 36°8 " |
| ——— frogs, - | a trace. | | 9° " |

If these results are confirmed, it will appear that the ingestion of phosphate of lime is not only indispensable for the reparation of bone, but that it is connected with the function of calorification.

In the second portion of his memoir, M. Mouries, starting from the principle demonstrated by Chossat, verified by Boussingault, taught by Berard, and now admitted by all physiologists, that diet is defective which does not contain enough phosphate of lime to repair the waste which is continually going on in the economy, attempts to prove that the food commonly consumed in cities does not contain the quantity of this salt which is required by nurses and pregnant women.

He commences by calculating the quantity of phosphate of lime which ought to be ingested in the twenty-four hours, which he estimates from analyses of the excreta at 110 grains. He then attempts to show that this quantity is not contained in the food of nurses in cities. The urine of women in the country contains 90 grains of phosphate of lime in the twenty-four hours, while the amount of this salt in the urine of women in cities varies from 20 to 90 grains. M. Mouries has sought to confirm his hypothesis by direct proofs; he has examined the food consumed in cities and shown that it exhibits a deficiency of one half in alkaline phosphates. He has examined the milk of nurses, and shown that in 18 healthy country women the proportion of earthy phosphates in the milk varied from 1.2 to 2 4 *per cent.*, while in the milk of ten Paris nurses the proportion varied from 0 5 to 0 9, and in seven others there was only a trace of phosphate of lime.

In the third portion of his essay M. Mouries adduces clinical facts in illustration of the advantage of supplying this deficiency of phosphate of lime in aliments. In 13 cases, in which the proportion of phosphate of lime averaged 0.7, 75 grains of this salt with twice that quantity of albumen was daily administered in soup; in a week the proportion of earthy phosphate in the milk rose to 2.1. In five cases pregnant women were subjected to the same treatment; the milk, after delivery, contained 1.9 to 2.1 of phosphate of lime. Only three of the eighteen children died.

These results, though insufficient to determine such a serious question, are yet very worthy of attention. In the debate to which they gave rise, M. Gibert vehemently condemned the present tendency of chemists to interfere in medical inquiry. The question of lactation was a medical one, he said, and was only to be solved by clinical observation. M. Bouchardt, on the other hand, feared only ignorance, and was not alarmed at the application of chemistry to medicines, especially when its results were as inoffensive as those he had discussed.

